# Single-cell mapping of maternal–fetal cross-talk in preeclampsia

**DOI:** 10.21203/rs.3.rs-8254581/v1

**Published:** 2025-12-08

**Authors:** Seungbaek Lee, Roberto Romero, Adi L. Tarca, Jose Galaz, Yi Xu, Gaurav Bhatti, Sivani Ravindran, Dustyn Levenson, Eva Kareus, Marcelo Farias-Jofre, Bogdan Panaitescu, Eloise Eyestone, Sonia S. Hassan, Tinnakorn Chaiworapongsa, Nandor Gabor Than, Roger Pique-Regi, Nardhy Gomez-Lopez

**Affiliations:** 1 Center for Reproductive Health Sciences, Department of Obstetrics and Gynecology, Washington University School of Medicine; St. Louis, MO, 63110, USA.; 2 Pregnancy Research Branch, Division of Obstetrics and Maternal-Fetal Medicine, Division of Intramural Research, *Eunice Kennedy Shriver* National Institute of Child Health and Human Development, National Institutes of Health, U.S. Department of Health and Human Services (NICHD/NIH/DHHS); Bethesda, MD, 20892, USA.; 3 Department of Obstetrics and Gynecology, University of Michigan; Ann Arbor, MI, 48109, USA.; 4 Department of Epidemiology and Biostatistics, Michigan State University; East Lansing, MI, 48824, USA.; 5 Center for Molecular Medicine and Genetics, Wayne State University School of Medicine; Detroit, MI, 48201, USA.; 6 Department of Obstetrics and Gynecology, Wayne State University School of Medicine; Detroit, MI, 48201, USA.; 7 Division of Obstetrics and Gynecology, School of Medicine, Faculty of Medicine; Pontificia Universidad Catolica de Chile; Santiago, Chile.; 8 Wayne State University School of Medicine; Detroit, MI, 48201, USA.; 9 Department of Pathology and Immunology, Washington University School of Medicine; St. Louis, MO, 63110, USA.; 10 Division of Maternal-Fetal Medicine, Department of Obstetrics, Gynecology and Women's Health, University of Missouri, Columbia, Missouri, 65212, USA.; 11 Office of Women's Health, Wayne State University; Detroit, MI, USA.; 12 Department of Physiology, Wayne State University School of Medicine; Detroit, MI, USA.; 13 Systems Biology of Reproduction Research Group, Institute of Molecular Life Sciences, HUN-REN Research Centre for Natural Sciences, H-1117, Budapest, Hungary.; 14 Department of Obstetrics and Gynecology, Medical School, Semmelweis University, Budapest, H-1088, Hungary.; 15 Department Maternity Private Clinic of Obstetrics and Gynecology, Budapest, H-1126, Hungary.

## Abstract

Preeclampsia (PE) arises from placental dysfunction, yet the cellular events that distinguish early-onset PE (EOPE) from late-onset PE (LOPE) are poorly defined. We generated a comprehensive single-cell atlas of the placenta of women with and without PE (78 patients), integrating maternal–fetal genotyping, multi-dimensional clustering, and cell–cell communication analysis. PE reshaped the abundance and transcriptional states of major placental cell types. EOPE was defined by expanded T and NK cells with reduced extravillous trophoblasts, whereas LOPE involved increased monocytes/macrophages and activation of ANNEXIN-, NOTCH-, ANGPTL-, and EGF-associated pathways. These onset-specific disruptions reveal distinct modes of immune–trophoblast dysregulation. Extending these insights to maternal circulation, multiple EOPE- and LOPE-derived placental mRNA signatures distinguished EOPE from controls at or after diagnosis, and an EOPE-derived CTB-1 protein signature showed moderate predictive value for LOPE. This atlas defines the cellular architecture of PE subtypes and establishes a foundation for developing early, noninvasive biomarkers.

Preeclampsia (PE) is a pregnancy-specific disorder characterized by new-onset hypertension (≥140/90 mmHg) and proteinuria after 20 weeks of gestation, affecting approximately 2–8% of pregnancies wordwide^1-4^. It remains a leading cause of maternal morbidity and mortality^2, 5^—accounting for up to 12% of maternal deaths during delivery in the United States^6^—and contributes substantially to fetal growth restriction, distress, and perinatal death^7, 8^. Clinically, PE is classified by gestational timing: cases diagnosed before 34 weeks are referred to as early-onset (EOPE) and those after 34 weeks as late-onset (LOPE)^9-12^. The only definitive treatment for PE is delivery of the placenta, which often necessitates a medically-indicated preterm delivery^1, 13, 14^. Despite its clinical significance, the cellular and molecular mechanisms that drive PE are poorly understood.

The pathogenesis of PE has been linked to abnormal placentation^15, 16^, defective remodeling of the spiral arteries^15-17^, impaired uteroplacental perfusion^18^, and syncytiotrophoblast stress responses^4^, ultimately provoking a systemic maternal inflammatory syndrome^15, 19, 20^. These processes underscore that the placenta—not the maternal vasculature alone—is central to disease initiation^1^. Yet, the molecular dialogue between maternal and fetal compartments within this organ is incompletely defined. Previous studies, including our own, have applied single-cell RNA sequencing (scRNA-seq) to map maternal–fetal interactions in normal pregnancy^21-28^ and to explore cellular alterations in PE^29-39^ (Supplementary Table 1). However, existing datasets have been limited by small cohort sizes, incomplete stratification of PE onset timing, and low resolution of immune cell diversity.

To overcome these limitations, we generated the most comprehensive single-cell atlas of PE to date, encompassing 39 placental samples spanning both early- and late-onset diseases as well as 39 gestational age (GA)–matched controls (n=78 total). This resource integrates maternal–fetal genotyping, high-resolution clustering, and crosscompartmental analyses to define the immune and stromal architecture of the placenta with unprecedented granularity. By combining pathway enrichment and cell–cell communication analyses, this atlas establishes a framework to dissect the signaling networks that connect structural and immune cells, establishing a foundation for discovering how cellular interactions at the maternal–fetal interface become perturbed in PE.

Recognizing the need to connect mechanistic discovery with clinical relevance, we further designed this work to evaluate the translational potential of single-cell signatures derived from placental tissues. Specifically, we aimed to determine whether scRNA-seq signatures derived from placental cell types from either EOPE or LOPE were detectable in the maternal circulation, and whether such signatures could ultimately inform predictive and diagnostic strategies for PE. To this end, we established an analytic framework capable of bridging single-cell data with circulating transcriptomic and proteomic profiles, laying the groundwork for the development of biomarker-based screening tools.

## Results

### A single-cell atlas of the human placenta in PE

**Fig. 1.**
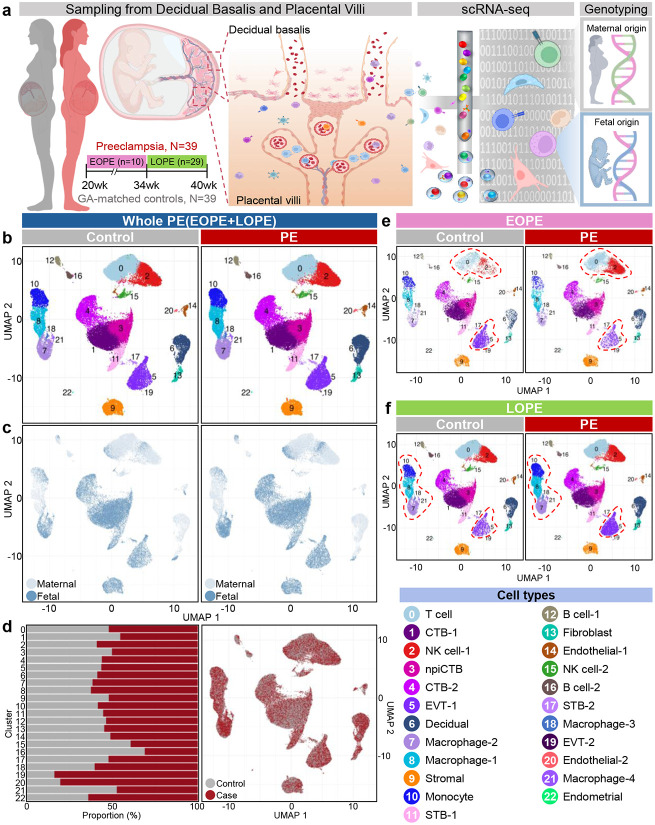
Single-cell atlas of the maternal-fetal interface in women with PE. (**a**) Study design illustrating the workflow from placental tissue collection (decidua basalis and placental villi) in women with PE (n = 39) and gestational age (GA)-matched controls (n = 39) for scRNA-seq. Maternal and fetal origins were assigned by genotyping. Uniform manifold approximation and projection (UMAP) plots show all cell types in (**b**) whole PE and controls. (**c**) The same UMAP embeddings are colored by maternal (light blue) or fetal (dark blue) origin. (**d**) Comparison of cell type abundance between whole PE cases and controls. (Left) Bar plot showing relative proportions of each cell type and (Right) UMAP plot colored by presence or absence of PE. The whole PE population was further stratified by PE onset timing into (**e**) EOPE and EOPE controls and (**f**) LOPE and LOPE controls.

To construct a comprehensive single-cell atlas of the human placenta from women with (n = 39) and without PE (n = 39) (Supplementary Table 2), decidua basalis and placental villi were utilized for scRNA-seq, and genotyping was conducted to assign maternal or fetal cellular origin using an established protocol^40^ (Fig. 1a, Extended Data Fig. 1). Within this atlas, unsupervised clustering analysis revealed 23 distinct cell types (Fig. 1b). Clusters included nonimmune cell types, such as syncytiotrophoblasts (STBs), cytotrophoblasts (CTBs), nonproliferative interstitial CTBs (npiCTBs), extravillous trophoblasts (EVTs), decidual cells, stromal cells, fibroblasts, endothelial cells, and endometrial cells. Clusters also included immune cell types, such as T cells, natural killer (NK) cells, macrophages, monocytes, and B cells. Trophoblast cells, as expected, were of fetal origin, along with some immune cell clusters such as Macrophage-2 (cluster 7) (Fig. 1b-c). Decidual and endometrial cells were, as expected, of maternal origin, as were most immune cell populations (Fig. 1b-c). Notably, PE affected cellular abundance in all placental cell types, regardless of origin (Fig. 1d, Supplementary Fig. 1). To assess differences in cellular composition depending on onset timing of PE, we stratified samples into EOPE and LOPE (Fig. 1e-f). In EOPE, EVT-1 (cluster 5) showed a two-fold reduction in abundance, whereas T cell and NK cell-1 clusters (clusters 0 and 2) showed increases, ranging from approximately 2-fold to 5.5-fold compared to GA–matched controls (highlighted by red dashed lines) (Fig. 1e, Supplementary Fig. 1). In LOPE, EVT-1 (2.4-fold), Monocyte (cluster 10) (~1.5-fold), and all macrophage clusters (clusters 7, 8, 18, and 21, with fold-changes up to 1.8) were increased compared to GA–matched controls (Fig. 1f, Supplementary Fig. 1). Thus, this single-cell atlas reveals that PE onset timing differentially reshapes the cellular composition of the human placenta.

### Immune-cell atlas of PE

**Fig. 2.**
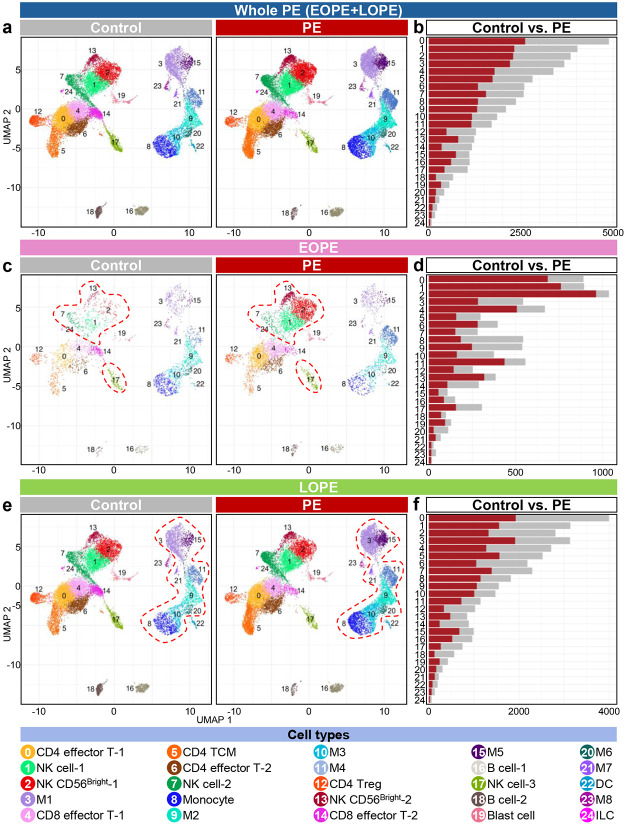
Immune cell atlas of the maternal-fetal interface in women with PE. UMAP plots and bar plots show immune cell subtype distributions and the number of each cell type in (**a-b**) whole PE and controls, (**c-d**) EOPE and EOPE controls, and (**e-f**) LOPE and LOPE controls. M, macrophage; TCM, central memory T cell; Treg, regulatory T cell; DC, dendritic cell; ILC, innate lymphoid cell

To explore the differential abundances of placental immune cell populations in control and PE, we subclustered immune cells into 25 distinct clusters (Extended Data Fig. 2), including T cell subtypes (clusters 0, 4, 5, 6, 12, and 14), NK cell subtypes (clusters 1, 2, 7, 13, and 17), macrophage subtypes (clusters 3, 9, 10, 11, 15, 20, 21, and 23), B cell subtypes (clusters 16 and 18), blast cells (cluster 19), and innate lymphoid cells (ILC; cluster 24) (Fig. 2a). Across all immune clusters, PE was associated with shifts in cell type abundance relative to controls (Fig. 2b).

Stratification by onset timing revealed distinct immune signatures. In EOPE, all NK cell clusters showed increased abundance compared to controls, with fold-changes of approximately 1.2 to 13.2 (highlighted cell types with the red dashed lines) (Fig. 2c, d). In LOPE, Monocyte (cluster 8) (1.6 fold) and all macrophage clusters except for M8 were increased relative to controls, with fold-changes up to 2.0 (Fig. 2e, f). Together, these data define an immune cell atlas of PE, demonstrating onset-specific remodeling of placental immune composition.

### Onset-dependent transcriptomic activity in PE

**Fig. 3.**
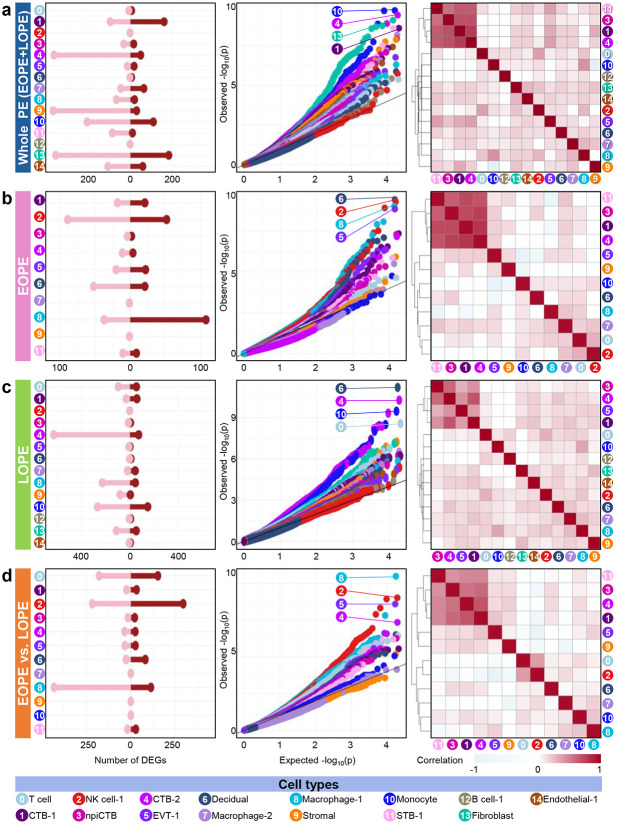
Transcriptomic profiles in the maternal–fetal interface associated with PE. Differentially expressed genes (DEGs) associated with PE were analyzed in (**a**) whole PE versus controls, (**b**) EOPE versus EOPE controls, (**c**) LOPE versus LOPE controls, and (**d**) EOPE versus LOPE. Lollipop plots show the number of DEGs per cell type, with red indicating up-regulated genes and pink indicating down-regulated genes. Quantile-quantile (Q-Q) plots illustrate the extent of transcriptomic changes, where deviation from the 1:1 line (black) reflects enrichment of DEGs; the top four representative clusters are shown with their cluster numbers. Heatmap represents log_2_ (fold change) correlations among cell types, where red represents stronger positive correlations.

To investigate transcriptional changes associated with PE, we analyzed gene expression profiles across nonimmune and immune cell types, retaining 15 clusters after filtering based on cell abundance (Supplementary Fig. 2). In the whole PE population, Fibroblast (cluster 13), CTB-2 (cluster 4), Stromal (cluster 9), and Monocyte (cluster 10) clusters showed the largest numbers of differentially expressed genes (DEGs), with Monocyte and CTB-2 being most strongly impacted by PE (Fig. 3a). Affected cell types varied with PE onset timing. In EOPE, Macrophage-1 (cluster 8), NK cell-1 (cluster 2), and Decidual (cluster 6) clusters showed the largest DEG counts and were most strongly affected by PE (Fig. 3b). In LOPE, CTB-2 and Monocyte clusters showed the largest DEG counts; Decidual, CTB-2, and Monocyte clusters were most affected by PE (Fig. 3c). Using interaction analysis to separate the unique contribution of each PE subset, we compared EOPE and LOPE. As a result, Macrophage-1 and NK cell-1 clusters showed the largest DEG counts and were most strongly affected by the onset timing of PE (Fig. 3d). Trophoblast subtypes (clusters 1, 3, 4, 5, and 11) showed highly correlated transcriptomic profiles regardless of PE onset timing (Fig. 3a-d). These findings demonstrate that PE has differential impacts on transcriptomic activity depending on cell type and disease onset timing.

**Fig. 4.**
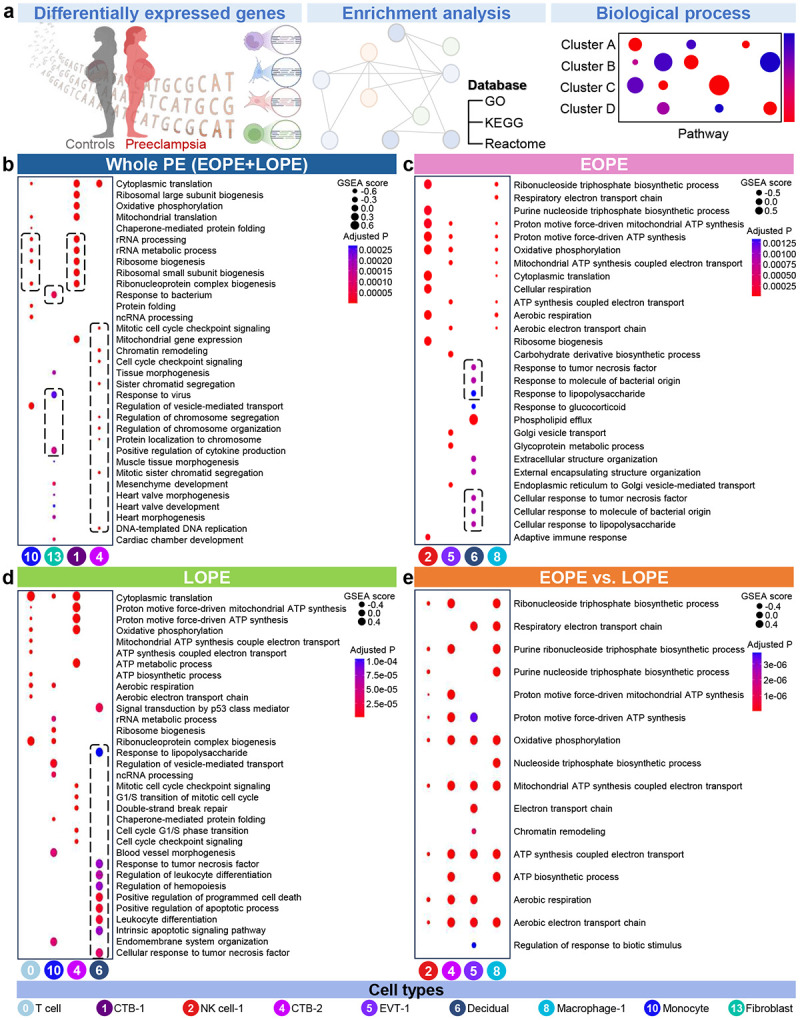
Pathway enrichment analysis of differentially expressed genes (DEGs). (**a**) Pathway enrichment analysis was performed using full ranked gene lists. ClusterProfiler dot plots show the top 20 enriched pathways for the four most affected cell types in (**b**) whole PE versus controls, (**c**) EOPE versus controls, (**d**) LOPE versus controls, and (**e**) EOPE versus LOPE. Dot size indicates gene set enrichment analysis (GSEA) score, while dot color represents statistical significance. Selected pathways of interest are indicated with dashed outlines.

Pathway enrichment analysis for the top four most affected clusters—those showing the greatest differential gene expression—from each population was conducted using full ranked gene lists based on Gene Ontology (GO), Kyoto Encyclopedia of Gene and Genomes (KEGG), and Reactome databases to identify biological processes associated with PE (Fig. 4a, Supplementary Table 3). In the whole PE population, cell-specific gene set enrichment analysis (GSEA) revealed processes related to cellular proteostasis in the Monocyte and CTB-1 (cluster 1) clusters, immune responses in the Fibroblasts cluster, and cell cycle regulation in CTB-2 (highlighted by the black dashed lines) (Fig. 4b). After stratifying the population based on PE onset timing, we found differential biological processes in the Decidual cluster. Specifically, in EOPE, the inflammatory response pathways were downregulated, whereas these pathways were upregulated in LOPE (Fig. 4c-d). However, specific immune cell types differentially responded between EOPE and LOPE (NK cell-1 and Macrophage-1 for EOPE and T cell (cluster 0) and Monocyte for LOPE) (Fig. 4c-d). Comparison between EOPE and LOPE revealed opposing regulation of biosynthetic processes across cell types, with these pathways downregulated in NK cell-1 but upregulated in Macrophage-1, EVT-1 (cluster 5), and CTB-2, highlighting that PE onset timing affects differential transcriptional responses (Fig. 4e). These results show that PE onset timing differently impacts biological processes in specific placental cell types.

### Cell–cell communication networks driven by PE-onset

**Fig. 5.**
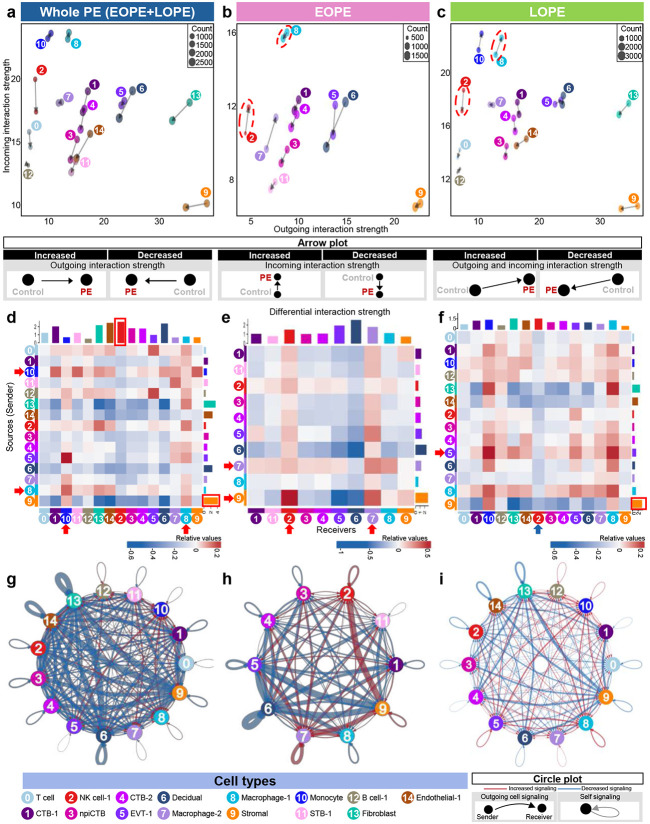
Cell-cell interactions in PE. Arrow plots showing changes in outgoing and incoming interaction strength between controls (arrowhead) and PE cases (arrow base) in (**a**) whole PE versus controls, (**b**) EOPE versus controls, and (**c**) LOPE versus controls. Heatmap illustrating differential interaction strength among cell types (senders and receivers) in (**d**) whole PE versus controls, (**e**) EOPE versus controls, and (**f**) LOPE versus controls. Red indicates increased signaling, and blue indicates decreased signaling in PE cases compared with controls. Circle plot showing the top 25% increased (red) or decreased (blue) signaling in (**g**) whole PE versus controls, (**h**) EOPE versus controls, and (**i**) LOPE versus controls.

We next investigated how PE-induced transcriptomic changes influence intercellular communication by mapping cell-cell interactions (Extended Data Fig. 3). Nonimmune cells, including trophoblasts (clusters 1, 3, 4, 5, and 11) and structural cells (clusters 6, 9, 13, and 14), generally decreased in incoming interactions in the whole PE group, as well as when we stratified by onset timing (Fig. 5a-c). However, immune cell types displayed distinct interaction patterns in EOPE and LOPE. Specifically, incoming interaction strength of NK cell-1 increased in EOPE but decreased in LOPE, and incoming and outgoing interaction strengths of Macrophage-1 decreased in EOPE but increased in LOPE. These cell types are emphasized with red dashed outlines in Fig. 5b and c. We next evaluated interaction strength between cell-type pairs (Fig. 5d-i). In the whole PE population, the Macrophage-1 and Monocyte clusters, highlighted by the red arrows, exhibited certain increased pairwise interactions with both nonimmune and immune cells compared to controls, whereas the Stromal and NK cell-1 clusters, emphasized by the red boxes, accounted for the highest overall interaction magnitudes as the predominant sender and receiver, respectively (Fig. 5d). In EOPE, the NK cell-1 and Macrophage-2 (cluster 7) clusters showed the strongest incoming interactions originating from the Stromal cluster (Fig. 5e, red arrows). Notably, Macrophage-2 also showed self-signaling loops indicative of positive feedback (Fig. 5e, red arrows). In LOPE, EVT-1 showed the most pronounced increase in outgoing signaling activity targeting both nonimmune and immune cells, although the Stromal cluster emerged as the dominant signal sender (Fig. 5f, red arrows and box). Notably, incoming signaling to NK cell-1 in LOPE was diminished (blue arrow), while in EOPE it was increased (red arrow) (Fig. 5f vs. 5e) indicating that disease onset differentially alters immune responses in the placenta.

Circle plots representing incoming and outgoing cell-cell signaling showed that, in whole PE, structural cells (e.g., Decidual, Stromal, Fibroblast, and Endothelial-1 clusters) underwent decreased signaling (Fig. 5g). When stratifying by disease onset, we observed increased outgoing signaling by NK cell-1, Macrophage-2, and Stromal clusters in EOPE, as well as increased self-signaling loops (Fig. 5h). By contrast, in LOPE, most cell-cell signaling was downregulated, as observed in the whole PE group (Fig. 5i).

**Fig. 6.**
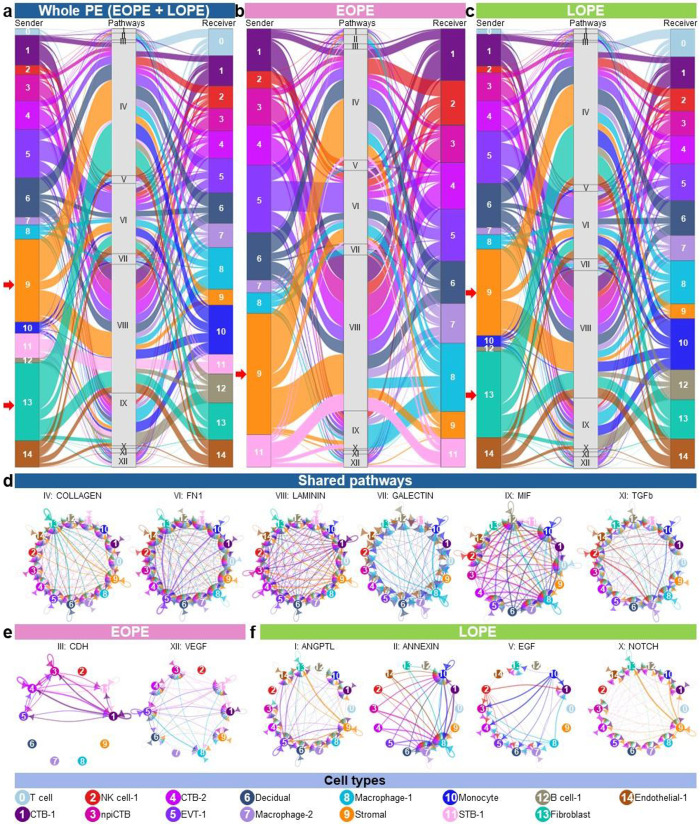
Top 25% aggregated intracellular communication in PE. Alluvial plots showing major outgoing signals from sender cells to signaling pathways and incoming signals to receiver cells in signaling pathways. (**a**) Whole PE, (**b**) EOPE, and (**c**) LOPE. The interactions show the contribution of cells in sending and receiving signals. Circle plots showing the significant cell-to-cell communications with probability > 0.9 for each signaling pathway. (**d**) Shared signaling pathways in whole PE, (**e**) only significant pathways unique to EOPE, and (**f**) only significant pathways unique to LOPE. Each node represents cell type, and connecting lines are color-coded based on the sender cell.

To delineate major communication processes and contributing cell types, we next focused on the top 25% of aggregated pathways affected by PE (Fig. 6, Supplementary Fig. 3). From these analyses, we visualized pathways that were consistently detected regardless of PE onset timing as well as those that differed greatly between EOPE and LOPE (Fig. 6a-c). Within these pathways, the Stromal and Fibroblast clusters served as the main senders, while immune cells functioned mainly as receivers (Fig. 6a). Yet, after stratifying, it was clear that the main sender in EOPE was the Stromal cluster, whereas both the Stromal and Fibroblast clusters were the prominent senders in LOPE (Fig. 6b, c). To display more detailed cell-cell communications for each signaling pathway, we visualized the major signaling pathways using circle plots (Fig. 6d-f). Signaling pathways observed regardless of PE onset timing included ECM remodeling (COLLAGEN, FN1, and LAMININ) and immune modulation (GALECTIN, MIF, and TGFb) (Fig. 6d), indicating that both structural and immune pathways are actively engaged in placental responses to PE. EOPE-specific pathways included CDH and VEGF pathways, both of which were largely governed by trophoblast cell types (Fig. 6e). LOPE-specific pathways, including ANGPTL, ANNEXIN, EGF, and NOTCH, showed broader intercellular interactions, engaging more nonimmune and immune cell types than affected pathways in EOPE (Fig. 6f).

Collectively, these findings reveal that PE alters nonimmune and immune intercellular communication in the placenta, yet these interactions are distinctly regulated based on the disease onset timing.

### Single-cell mRNA signatures derived from placentas of women with PE are detectable in maternal circulation

Having established the single-cell atlas of PE, we next determined whether PE-derived single-cell mRNA signatures (hereafter referred to as PE-derived signatures) could be detected in maternal circulation (Supplementary Fig. 4a). These signatures were independently derived from each PE group—whole PE, EOPE, and LOPE—when compared to their respective controls (Supplementary Table 4). To monitor such signatures in maternal circulation, we utilized a longitudinal whole-blood microarray dataset which included samples from normotensive women^41^. The effect of gestational age was removed using linear mixed-effects models with non-linear terms of gestational age. The whole PE-derived signatures that significantly changed across gestation were those of T cell, CTB-1, and Endothelial-1 (Supplementary Fig. 4b). The EOPE-derived signatures that significantly changed across gestation were those of CTB-1 and EVT-1 (Supplementary Fig. 4c). The LOPE-derived signatures that significantly changed across gestation were those of Macrophage-2, Macrophage-1, B cell-1, Fibroblast, and Endothelial-1 (Supplementary Fig. 4d). The PE-derived signatures that did not significantly change throughout gestation are shown in Supplementary Table 5. These data show that PE-derived signatures can be monitored throughout gestation, but their detectability differs based on onset timing.

### Single-cell mRNA signatures derived from placentas with PE are detectable at diagnosis and discriminate PE patients from controls

**Fig. 7.**
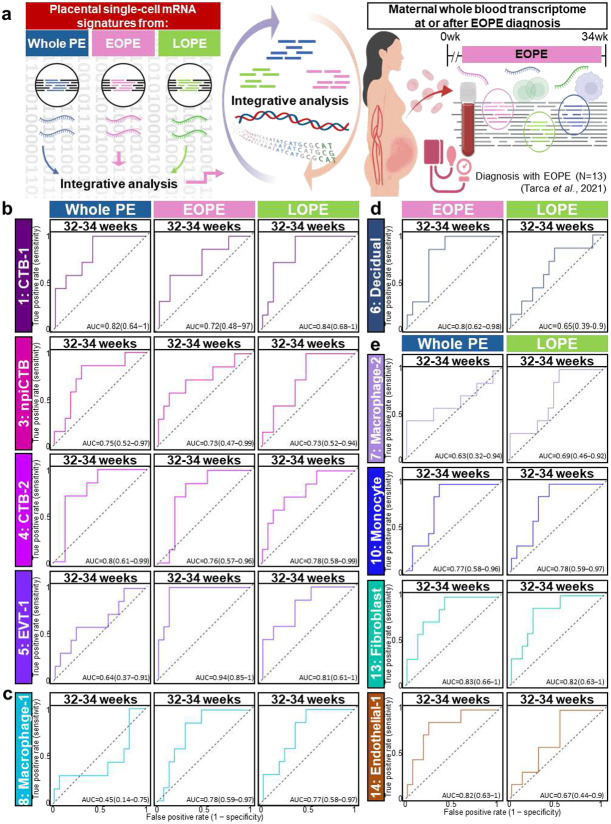
Placental single-cell signatures detected in maternal circulation at or after EOPE diagnosis. (**a**) Schematic overview of the integrative analysis between placental single-cell signatures and whole-blood transcriptomic profiles from EOPE cases (n = 13) at or after EOPE diagnosis. ROC curves showing the discriminative performance for EOPE and controls of (**b**) trophoblast cell types, (**c**) Macrophage-1, (**d**) Decidual, (**e**) other immune and structural cell type-derived signatures. AUC values with 95% confidence intervals are reported for each signature, with values > 0.5 indicating discrimination above chance.

Next, we investigated whether PE-derived signatures can be detected in the maternal circulation at or after EOPE diagnosis. To test this, we assessed our PE-derived signatures in a whole-blood microarray gene expression dataset, which included longitudinal blood samples from women who were ultimately diagnosed with EOPE alongside GA-matched controls^41^. Receiver operating characteristic (ROC) curves were generated for each signature (derived from either whole PE, EOPE or LOPE), and an area under the curve (AUC) values were calculated (Fig. 7, Supplementary Table 6). Notably, all PE-derived signatures from trophoblast cell types discriminated EOPE from controls at 32-34 weeks, regardless of whether they were derived from placentas from women with EOPE or LOPE (Fig. 7b). Similarly, EOPE-derived (AUC = 0.78) and LOPE-derived (AUC = 0.77) signatures from the Macrophage-1 cluster were able to discriminate between EOPE and controls, whereas the whole PE-derived signature performed poorly (AUC = 0.45) (Fig. 7c). The Decidual signature derived from EOPE (AUC = 0.8) could differentiate EOPE from controls more effectively than the LOPE-derived signature (AUC = 0.65) (Fig. 7d). However, not all EOPE- or LOPE-derived signatures could discern EOPE from controls in blood samples (Supplementary Table 6). For example, LOPE-derived signatures from Macrophage-2 (AUC = 0.69), Monocyte (AUC = 0.78), Fibroblast (AUC = 0.82), and Endothelial-1 (AUC = 0.67) could discriminate between EOPE and controls (Fig. 7e), but not if they were derived from EOPE (Supplementary Table 6).

We further validated these results using another cross-sectional whole-blood microarray dataset, which included samples collected from women diagnosed with either EOPE or LOPE and their controls^42^ (Supplementary Fig. 5a, Supplementary Table 7). Gestational age was explicitly adjusted by regressing each gene on gestational age within controls and using residuals as normalized expression values. This analysis confirmed that PE-derived signatures are also detectable in maternal circulation at the time of LOPE diagnosis as well and able to discriminate PE patients from controls (Supplementary Fig. 5b-d). Collectively, these data indicate that mRNA signatures derived from EOPE or LOPE placentas may serve as biomarkers capable of discriminating PE patients from controls.

### Protein signatures derived from specific cytotrophoblasts display predictive value for LOPE

**Fig. 8.**
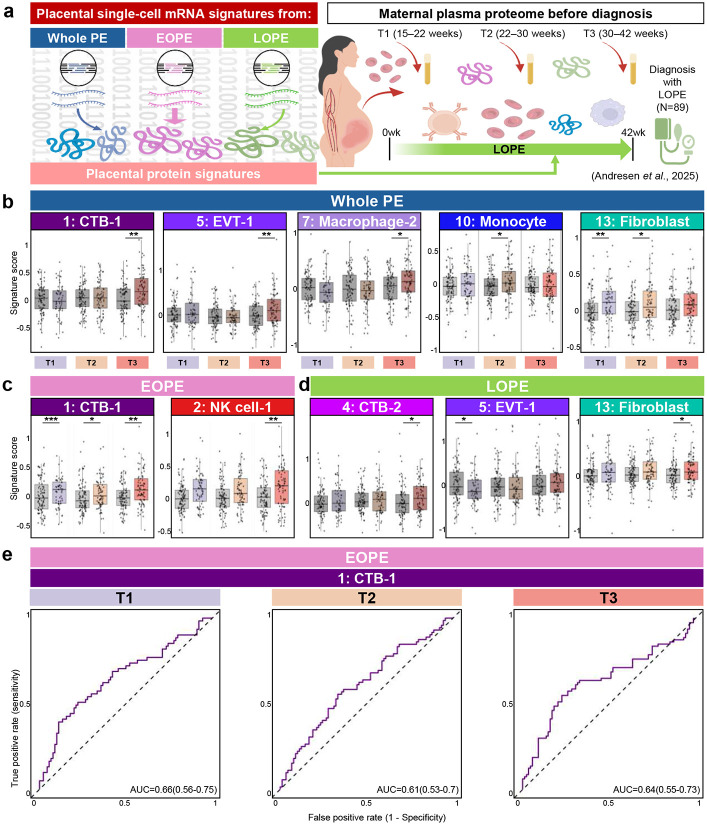
Proteomic validation of placental single-cell transcriptomic signatures in maternal plasma. (**a**) Schematic overview of the integrative analysis assessing whether mRNA-level placental single-cell signatures translate into protein-level changes detectable in maternal plasma from LOPE cases (n = 89). (**b**) Boxplots showing protein-level abundance corresponding to cell type signatures derived from (**b**) whole PE, (**c**) EOPE, and (**d**) LOPE analyses across three gestational windows: T1 (15–22 weeks), T2 (22–30 weeks), and T3 (30–42 weeks). (e) ROC curves showing the predictive performance of CTB-1 signatures from EOPE. AUC values with 95% confidence intervals are reported for each time interval, with values > 0.5 indicating prediction above chance.

Because mRNA-based biomarkers can be challenging to implement in low-resource settings^43, 44^, and given our previous reports on the relevance of placenta mRNA signatures to proteomics profiling in amniotic fluid and blood^45^, we next quantified EOPE- and LOPE-associated placental protein signatures based on corresponding single-cell mRNA signatures across gestational windows (T1; 15-22 weeks, T2; 22-30 weeks, and T3; 30-42 weeks) (Fig. 8a). A longitudinal plasma proteomic dataset from women with normal pregnancy and those who ultimately developed LOPE was used for this analysis^46^. The effect of gestational age was removed by fitting generalized additive models.

Proteomic profiling revealed that increased protein signature abundance in LOPE was not limited to trophoblast-derived signatures but also extended to immune and structural cell signatures (Fig. 8b). Among the whole PE-derived signatures, Fibroblast signatures were significantly elevated during T1 (p = 0.004) and T2 (p = 0.01), while trophoblasts and Macrophage-2 signatures showed the most pronounced increases during T3 (p_CTB-1_ = 0.001, p_EVT-1_ = 0.007, p_Macropahge-2_ = 0.01). Stratification by onset timing highlighted distinct, onset-specific signatures. Notably, the EOPE-derived CTB-1 signatures showed a significant increase in abundance and remain consistently higher across all gestational windows (p_T1_ = 0.001, p_T2_ = 0.01, p_T3_ = 0.02) (Fig. 8c). By contrast, the LOPE-derived trophoblast signatures emerged from CTB-2 and EVT-1 with significant increases during T3 and T1, respectively (p_CTB-2_ = 0.04, p_EVT-1_ = 0.04) (Fig. 8d). Other EOPE- and LOPE-derived protein signatures showed variable case–control differences depending on gestational timing (Supplementary Table 8). We next evaluated the predictive performance of the EOPE-derived CTB-1 protein signature and found that it achieved moderate predictive accuracy across gestational windows (Fig. 8e). Taken together, these findings indicate differential mRNA translation across EOPE and LOPE, highlighting the need for biomarkers tailored to disease onset timing and nominate cytotrophoblast-associated factors as leading candidates for the development of a protein-based point-of-care test to identify women at risk of PE.

## Discussion

In this study, we generated the most comprehensive single-cell atlas of the human placenta and decidua in PE to date, integrating disease onset timing, maternal–fetal origin, cellular abundance, and intercellular communication. This multi-dimensional resource reveals that PE profoundly reshapes the placental landscape at both the cellular and molecular levels, and that EOPE and LOPE represent biologically distinct entities with divergent immune, trophoblast, and stromal perturbations. By extending these single-cell signatures to maternal blood, we further demonstrate that placental molecular states can be traced systemically during pregnancy, nominating cell type–specific placental signatures as promising candidates for biomarker discovery.

EOPE is strongly linked with early placental defects – including insufficient EVT invasion^15, 16^, inadequate spiral artery remodeling^15-17^, and impaired uteroplacental perfusion^18^ - which collectively contribute to fetal growth restriction^47^. By contrast, LOPE is thought to arise from maternal microvascular^19^ or metabolic disease^19, 48^ and is characterized by a lower incidence of fetal growth restriction^47, 49^, largely preserved spiral artery remodeling^20, 47, 50^, and normal umbilical artery blood flow^47^. A major insight from our dataset is that EOPE and LOPE exhibit distinct patterns of cellular dysregulation. EOPE was characterized by increased placental T cells and NK cells and reduced EVT-1 abundance. These findings parallel previous reports of expanded effector T cell populations at the maternal–fetal interface in women with PE, particularly in EOPE^51, 52^, as well as increased activated and tissue-resident memory T cells in EOPE decidua^53^, and reduced proportions of regulatory T cells in PE^51, 52, 54^. Our pathway and cell–cell communication analyses, particularly reduced VEGF signaling, further suggest that increased NK cell activity in EOPE may contribute to EVT depletion, consistent with studies showing that EOPE-associated decidual NK cells impair trophoblast invasion and promote trophoblast lysis through elevated IFN-γ, perforin, and granzyme B expression^55^. Additionally, decidual cells emerged one of the most affected cell types by PE and showed downregulated inflammatory response pathways, aligned with previous findings of diminished inflammation-related signaling in decidualized cells^56^. As key immune modulators at the maternal-fetal interface, decidual cells regulate the proliferation and functional maturation of immune populations, including T cells and NK cells. For example, galectin-1, highly expressed in decidual cells^57, 58^, modulates T-cell proliferation^59^, survival^59, 60^, and the production of proinflammatory cytokines^59, 61^. Moreover, decidual cells promote the acquisition of the low-cytotoxic phenotype of decidual NK cells^61-63^. Therefore, a deficient decidual environment, often referred to as decidualization resistance^64, 65^, may impair both stromal differentiation and local immune regulation. Together, these data suggest a mechanism in which amplified cytotoxic signaling promotes trophoblast apoptosis, reducing trophoblast abundance and ultimately limiting their invasion into the decidua.

In contrast, LOPE was defined by increased monocytes and macrophages, with maternal macrophages emerging as major receivers in onset-specific signaling networks. These findings align with studies implicating macrophage plasticity and inflammatory activation in PE pathophysiology^66-69^ and with the prominent involvement of macrophages in acute atherosis^70, 71^, a vascular lesion commonly observed in PE^71, 72^. LOPE-specific cell–cell communication networks were enriched for ANNEXIN, EGF, NOTCH, and ANGPTL signaling pathways. Annexin family proteins are expressed across trophoblast, decidual, and immune compartments^73^, promote trophoblast invasion^74-76^, and mediate thrombomodulatory functions essential for maintaining placental blood flow^77-79^. Altered annexin expression has been reported in both placentas^76, 79-81^ and maternal circulation^78, 79, 82-84^ of women with PE and is associated with inflammatory responses^82, 85, 86^, oxidative stress–induced trophoblast death^87-89^, and microvascular thrombosis^78, 79, 90^. Our findings reinforce the potential contribution of annexin-associated pathways to LOPE and highlight the need to dissect cell type–specific functions of annexin signaling in placental dysfunction.

Our atlas also reveals how PE onset timing reshapes intercellular communication. Structural nonimmune cell types exhibited globally reduced incoming signal strength in both EOPE and LOPE, whereas immune cell populations displayed strikingly divergent behaviors. In EOPE, NK cell-1 and Macrophage-2 acted as major signaling hubs, whereas in LOPE, Macrophage-1, Monocyte, and B cell-1 mediated the strongest signaling. These findings underscore the biological distinction between EOPE and LOPE and illustrate how timing-dependent remodeling of trophoblast–immune communication contributes to their divergent pathophysiology.

Early identification of PE is essential to prevent the increased risk of progression to severe multisystem disorders, including eclampsia (seizure)^91, 92^, HELLP syndrome (hemolysis, elevated liver enzyme levels, and low platelet levels)^93^, cardiovascular disease^5, 94-96^, and stroke^97, 98^. Additionally, timely detection enables early monitoring and clinical intervention, thereby mitigating maternal and fetal morbidity and ultimately reducing morality during pregnancy and the postpartum period. Over the past decade, numerous screening strategies have been evaluated to improve PE prediction^99^. For example, Doppler sonography in combination with biochemical markers, such as placental growth factor (PlGF) and glycoprotein pregnancy-associated plasma protein-A (PAPP-A), has been widely used^100-102^. Additionally, several circulating biomarkers, including soluble fms-like tyrosine kinase-1 (sFlt-1)^103^, soluble endoglin (sEng)^104, 105^, β-human chorionic gonadotropin (β-hCG)^106^, a-disintegrin and metalloprotease 12 (ADAM-12)^107, 108^, matrix metalloproteinase 7 (MMP-7)^101^, placental protein actibin-A^102^ and inhibin-A^109-111^ , and microRNAs^112^ have shown promise. However, despite alterations of these markers in women with PE compared to normotensive controls, several limitations hinder their clinical application: their relationship to PE pathophysiology is not always clear, predictive performance is modest, and no consensus guidelines support their routine use in clinical practice. These limitations underscore the need for biomarkers that directly reflect the underlying cellular and molecular perturbations driving PE. To address this gap, we leveraged scRNA-seq to define mRNA signatures with cellular origins. We then evaluated the translational potential of these signatures across different datasets, thereby linking placental cell dysfunction to detectable circulating signals. Using this integrative approach, we reported that PE-derived signatures are detectable in the maternal circulation and even able to distinguish between PE cases and controls. Additionally, we showed onset-specific protein signatures, among which CTB-linked factors emerged as promising candidates for translation into clinically implementable assays. While the pathophysiology of EOPE and LOPE are distinct, the two subtypes share common features such as placental oxidative stress and placental dysfunction. For example, in LOPE, various factors can contribute to increased placental oxidative stress, including placental hypoperfusion arising from abnormal placentation in early pregnancy^113^, increased metabolic demands of the placenta and fetus^4, 114^, and maternal predisposition to cardiovascular disease^114^. Additionally, a subset of LOPE exhibited oxidative and endoplasmic reticulum stress profiles similar to those observed in EOPE cases^115^. These shared pathological pathways between EOPE and LOPE provide a rational for the detection of EOPE-derived trophoblast signatures in plasma samples collected from LOPE cases. However, further experimental and clinical validation is needed to verify the accuracy and translational relevance of these prediction markers.

As placental signaling networks are highly complex, future efforts that integrate multi-omic datasets with machine-learning–based approaches^116-121^ may further refine predictive models and support development of a clinically deployable point-of-care test for PE. Together, our findings provide a mechanistic, cell type–resolved framework for understanding how PE arises and diverges into early and late subtypes. By coupling mechanistic insights with biomarker discovery, this work establishes a foundation for defining disease pathways, prioritizing therapeutic targets, and developing noninvasive diagnostic strategies.

This study has several considerations that also outline important opportunities for future research. First, transcriptomic signatures derived from placental tissues collected at delivery may not fully capture the dynamic molecular changes that precede the clinical onset of PE. Nonetheless, our comprehensive atlas—capturing onset timing, maternal–fetal origin, and cross-compartmental signaling—provides a crucial reference point for future studies aimed at defining early disease drivers. Second, single-cell approaches are inherently influenced by dissociation- and sequencing-related biases, and certain populations—such as large trophoblast subtypes (e.g., STB) and fragile immune cells (e.g., neutrophils)—may be under-represented due to technical constraints^40^. However, comparative analysis revealed strong concordance between expression changes quantified by microarray analysis of placental bulk^122^ and those detected in our scRNA-seq dataset, including across trophoblast clusters such as STB (Supplementary Fig. 6). This cross-platform agreement indicates that core transcriptional perturbations in PE are robustly captured despite partial underrepresentation of specific cell types. Third, while our integrative analyses identified PE-associated mRNA signatures with promising predictive potential for EOPE and LOPE, additional experimental validation in large, diverse, and prospectively collected cohorts will be essential to determine their clinical utility.

Taken as a whole, our work delineates the cellular origins, signaling disruptions, and systemic molecular consequences of EOPE and LOPE at unprecedented resolution, demonstrating that placental single-cell states propagate into the maternal circulation and establishing a conceptual and practical foundation for future efforts aimed at mechanistic discovery, biomarker development, and early, noninvasive detection of the PE syndrome.

## Methods

### Study design

We conducted a prospective cross-sectional study of pregnant women with PE (n = 39) and gestational-age matched control without the diagnosis of PE (n = 39). PE was defined as new-onset hypertension and proteinuria after 20 weeks of gestation^3^. Hypertension was diagnosed when systolic or diastolic blood pressure reached at least 140 or 90 mmHg, respectively, measured on at least two separate occasions taken four hours to one week apart^3^. Proteinuria was defined as ≥300 mg of protein in a 24-hour urine collection, or two random urine specimens obtained four hours to one week apart showing protein ≥1+ by dipstick or one dipstick demonstrating ≥2+ protein^3^. Women diagnosed with PE were divided into those who developed EOPE (diagnosed and delivered before 34 weeks of gestation, n = 10) and LOPE (delivery at or after 34 weeks of gestation, n = 29). Pregnant participants without a diagnosis of PE were gestational age-matched to women diagnosed with PE. Women carrying twin pregnancies, or fetuses with chromosomal and/or sonographic abnormalities were excluded. Maternal and neonatal data were obtained by retrospective clinical chart review. Demographic and clinical characteristics of the study groups are shown in Supplementary Table 2. Placental tissue samples were collected at delivery from eligible women enrolled in our research protocols at the Detroit Medical Center, Wayne State University School of Medicine, and the Pregnancy Research Branch, an intramural program of the *Eunice Kennedy Shriver* National Institute of Child Health and Human Development, National Institutes of Health, US Department of Health and Human Services (NICHD/NIH/DHHS), Detroit, MI, USA. The collection and use of human materials for research purposes were approved by the Institutional Review Boards of Wayne State University and the NICHD (IRB Numbers: 031318MP2F and 061217MP2F). Before sample collection, written informed consent was provided by all participating women.

### Laboratory procedures

#### Sample collection

Immediately after the delivery of the placenta (within 30 minutes to up to 2 hours), the basal plate -including the decidua basalis- with placenta villi (BPPV) were collected as previously described^40^ and maintained in ice-cold 1X phosphate-buffered saline (PBS) (Thermo Fisher Scientific/Gibco) until the dissociation protocol was initiated. Maternal samples (peripheral blood or myometrium) and fetal samples (cord blood or umbilical cord tissue) were collected and stored at −80°C for subsequent genotyping of maternal and fetal DNA, respectively.

#### Preparation of single-cell suspensions from placental tissue

Immediately following placental tissue collection, the BPPV were mechanically and enzymatically homogenized to prepare single-cell suspensions following our previously established protocol^40^. Briefly, the BPPV were minced and enzymatically digested using the Umbilical Cord Dissociation Kit (Miltenyi Biotec) followed by incubation at 37°C. The resulting cell suspensions were washed with 1X PBS (Thermo Fisher Scientific), filtered through 100 μm cell strainers (Miltenyi Biotec), and centrifuged at 300 × g for 10 minuntes at room temperature. Erythrocytes were lysed with ACK lysis buffer (Life Technologies). Cells were washed with 1X PBS and then resuspended in 0.04% bovine serum albumin (BSA) (Sigma Aldrich) in 1X PBS and filtered through 30 μm strainers (Miltenyi Biotec). Cell concentration and viability were assessed using an automated counter (Cellometer Auto 2000, Nexcelom Bioscience), and the Dead Cell Removal Kit (Miltenyi Biotec) was used to remove dead cells to reach a viability of ≥80%.

#### Single-cell GEM generation and library construction

Single-cell RNA sequencing (scRNA-seq) libraries were prepared from viable cells using the 10x Genomics Chromium Single Cell 3’ Gene Expression Version 3.1 Kit (10x Genomics), following the manufacturer’s instructions. Briefly, single-cell suspensions were loaded onto the Chromium Controller to generate Gel Bead-in-Emulsions (GEMs), in which a single cell and a single Gel Bead with barcoded oligonucleotides were encapsulated. mRNA was reverse-transcribed into complementary (c)DNA using the Veriti 96-well Thermal Cycler (Thermo Fisher Scientific), and the resulting cDNA was purified using Dynabeads MyOne SILANE (10x Genomics) and the SPRIselect Reagent (Beckman Coulter). The size of cDNA amplicon was optimized by enzymatic fragmentation, end repair, and A-tailing. Next, adaptors and sample index were then ligated, and the PCR product was amplified using the Veriti 96-well Thermal Cycler. Double-sided size selection was performed with the SPRIselect Reagent kit, and the size and concentration of the final library construct were determined using the Agilent Bioanalyzer High Sensitivity DNA Chip (Agilent Technologies).

#### Library sequencing

The post-library constructs were quantified using the KAPA DNA Quantification Kit for Illumina platforms (Kapa Biosystems) before sequencing, according to the manufacturer’s protocol. Sequencing of the scRNA-seq libraries was performed by NovoGene (Sacramento, CA, USA) using the Illumina Platform (HiSeq X Ten System).

#### DNA isolation for genotyping

Genomic (g)DNA was extracted from blood or tissues using the DNeasy Blood and Tissue Kit (Qiagen), following the manufacturer’s protocols modified with the addition of 4 μL RNase A (100 mg/mL) (Qiagen) and incubation at 56 °C. Purified gDNA concentrations were measured using the Qubit^™^ dsDNA HS Assay Kit (Invitrogen). Genotyping was performed using two platforms: (i) low-coverage (~0.4X) whole-genome sequencing imputed to 37.5 M variants with the 1000 Genomes database (Gencove) and (ii) Infinium Global Diversity Array-8 v1.0 Kit microarrays processed by the Advanced Genomics Core of University of Michigan (Ann Arbor, MI, USA). For the array platform, genotype information was converted to vcf format using “iaap-cli gencall” and “gtc_to_vcf.py” from Illumina and imputed to 37.5M variants via the University of Michigan Imputation Server (https://imputationserver.sph.umich.edu/) with the 1000 Genomes haplotype references. Maternal/fetal relationships of the genotyped samples were confirmed using plink2 KING-robust kinship analysis^123^. The vcf files from both platforms were merged and filtered using bcftools for high-quality imputation and coverage for at least ten scRNA-seq transcripts.

### Data analysis

#### scRNA-seq data normalization and pre-processing

Raw sequencing output from Novogene was demultiplexed using Cell Ranger version 7.0.0 (10x Genomics). “cellranger count” was applied to align the sequencing reads using the STAR aligner^124^. For each library, quality metrics, including the average number of unique molecular identifiers (UMIs), the average number of detected genes, cell count, the average number of reads per cell, the fraction of reads in cells, percentage of reads mapping to the mitochondrial genome, and valid barcodes across the prepared libraries were computed. The bam files and the genotype vcf file were used for demultiplexing the individual of origin based on the genotype information using demuxlet^125^. Any droplet/GEM barcode assigned to doublets or ambiguous cells in demuxlet were removed, retaining only cells that could be assigned to a pregnancy case and to maternal/fetal origin were kept. Cells with less than 200 detected genes or with mitochondrial reads exceeding 25%, were further removed. All count data matrices were then normalized and combined using the “NormalizeData,” “FindVariableFeatures,” and “ScaleData” methods implemented in the Seurat package in R (Seurat version 5.0.1, R version 4.3.2)^126, 127^. The first 100 principal components were obtained using the Seurat “RunPCA” function, and the different libraries were integrated and harmonized using the Harmony package (version 1.0)^128^. The first 30 Harmony components were embedded and visualized into a two-dimensional map using the Seurat “RunUMAP” function, which implements the Uniform Manifold Approximation and Projection (UMAP) algorithm for dimensionality reduction^129, 130^. A resolution of 0.5 was used to cluster the single cells, which was selected after testing multiple values (0.2-1.0) and determining that it yielded the most interpretable clustering results.

#### Cell type annotation

Cell type annotation was performed using the SingleR package (version 1.4.1) in R^22, 25, 131^. SingleR calculates the Spearman correlation coefficients between the single-cell gene expression data and samples from the reference dataset using only variable genes. The multiple correlation coefficients per cell type are combined according to the cell type labels of the reference dataset to assign a score for each cell. In addition to these datasets, a 10x Genomics peripheral blood mononuclear cell (PBMC) dataset was used to annotate immune cells^132^. An anchor-based supervised mapping workflow was used to integrate reference and query single-cell datasets and assign cell type annotation based on the shared biological states using the FindTransferAnchors and MapQuery functions in Seurat version 4.0.1. The query dataset was normalized and scaled using the SCTransform function. Anchors between the query and reference datasets were then identified using supervised principal component analysis (sPCA)^133^, which select components that maximize dependence between datasets, quantified by the Hilbert-Schmidt Independence Criterion. Next, cell type labels were transferred using a binary classification model built on the reference annotations, and the nearest neighbors between reference and each query cell were identified. Final cell type labels were determined by majority vote across annotations from SingleR and Seurat, applied to four reference datasets^22, 25, 131, 132^. If multiple clusters were assigned to the same consensus cell type, a sub-index was added to distinguish the original Seurat cluster. For the immune cell-specific analysis, immune cell subclusters were extracted and reprocessed using the same procedures detailed in the previous section (“FindVariableFeatures”, “ScaleData”, “RunPCA”, Harmony and UMAP). 30 Harmony components were selected and a clustering resolution of 1.2 was used. The resulting clusters were re-annotated using the Azimuth PBMC reference (version 0.5.0)^127^ based on the level 2 annotations^132^. Annotation results from Azimuth were further validated by examining marker gene expression for each immune cell subset identified by FindAllMarkers (Wilcoxon rank–sum test) in Seurat. Marker genes were defined with the parameters min.pct = 0.25, logfc.threshold = 0.25, and only.pos = TRUE.

#### Differential gene expression in PE

DEGs associated with PE were identified using the DESeq2 package (version 1.32.0) in R^134^. First, we created a pseudo-bulk aggregate by adding the reads of all the cells of the same cell type and pregnancy sample, hereafter referred to as a combination. Only combinations with more than 20 cells per sample were analyzed, all others were treated as non-observed. Each combination represents an observed column in the data matrix provided to DESeq2 where each row represents a gene. The library identifier was added as a factor in the DESeq2 model to correct technical batch effects. Cell types found in less than three samples per study group for a given combination were excluded from this analysis. Differential expression was assessed using four independent DESeq2 analyses: i) whole PE vs. whole PE controls, ii) EOPE vs. EOPE controls, iii) LOPE vs. LOPE controls, and iv) an interaction model contrasting EOPE and LOPE. P-values were adjusted for multiple testing using the Benjamini–Hochberg false discovery rate (FDR), and DEGs were defined as those with an adjusted p-value (FDR) <0.1. Quantile–quantile plots were used to assess the distribution of the p-values and identify cell types enriched for low p-values.

#### Gene ontology and pathway enrichment analysis of genes affected by PE

Gene Set Enrichment Analysis (GSEA) was performed using the clusterProfiler package (version 4.0.4) in R^135^ based on the Gene Ontology (GO), Kyoto Encyclopedia of Gene and Genomes (KEGG), and Reactome databases. The functions “gseGO”, “gseKEGG”, “gsePathway” were applied separately to each ranked gene list obtained from the differential expression analysis for each cell type. Genes were ranked by – log_10_(p-value), and enrichment scores were calculated for each pathway. Results with an adjusted q-value < 0.05 were considered statistically significant.

#### Cell-cell communication analysis

Cell-cell communication analysis was performed using the CellChat R package (version 2.1.1) to infer cell-cell communications using the single-cell gene expression data and a database of prior knowledge of the interactions between signaling ligands, receptors, and their cofactors. The database integrates signaling molecule interaction information from KEGG and experimental studies, with curated interactions categorized into 299 signaling pathways. Significant communication between cell groups (clusters) is predicted by identifying over-expressed ligands and receptors between cell groups (p < 0.05). It calculates the communication probabilities for each ligand–receptor pair using a mass action-based model based on the average expression level of a ligand in the sender population and its cognate receptor(s) in the receiver population. This probability is further adjusted for the proportions of cells in each cell group, under the assumption that more abundant cell groups are more likely to send stronger signals than rare groups. In the estimated cell-cell communication network with weights as probabilities, centrality metrics from graph theory are applied to identify the major signaling roles. These analyses predict the key sending and receiving signals between specific cell groups. For the CellChat analysis, we only used cell types with at least 200 cells for each condition combination. The top 25% of significant cell-cell communications, ranked by communication probability, were selected across different pathways and visualized using the CellChat R package (version 2.1.1) and ggplot2 R package (version 3.5.1). The major sending and receiving signaling roles were determined based on context-specific pathways across different cell groups. The comparison between the overall information flow from the two study groups (PE versus control or EOPE versus LOPE) was performed using the paired Wilcoxon test with the function “rankNet” from CellChat.

#### Comparative analysis between placental single-cell and bulk transcriptomics

To assess the agreement between the single-cell-specific PE signatures derived in this study and prior reports in bulk placenta, we performed a correlation analysis of log2 fold changes (e.g. EOPE vs. EOPE control) observed in each cell type and log2 fold changes in bulk placenta analysis reported by Than et al., 2018^122^ between preterm severe PE, with or without HELLP (hemolysis, elevated liver enzymes, low platelets count) syndrome (n=12) and preterm controls (n=5) using Whole Human Genome Oligo Microarray G4112A (Agilent Technologies, Santa Clara, CA, USA). Spearman correlation of log2 fold changes and corresponding p-values were obtained using data from all genes with q<0.1 reported in Than et al., 2018^122^, regardless their significance status in each cell type in our study.

#### Longitudinal analysis of signatures in maternal whole-blood transcriptomes

Placental single-cell signatures were evaluated in maternal circulation using a longitudinal whole-blood microarray dataset from normotensive pregnancies^41^. Microarray probe intensities were standardized by subtracting the mean and dividing by the standard deviation of in control samples collected before 11 weeks gestation, yielding Z-scores. For each cell type, a per-sample signature score was calculated as the mean of the standardized expression Z-scores of the probes corresponding to the genes comprising the signature. Trends in cell-type scores vs gestational age were estimated using linear mixed-effects models (lme4 package) to account for longitudinal observations from the same subjects^136^. The models included, as fixed effects, a B-spline transformation of gestational age (degree 2) to capture non-linear trends and a random effect for each subject. A global significance test for any change with gestational age was performed using ANOVA function in lme4 package^136^.

#### Cross-sectional analysis of placental single-cell signatures in maternal whole-blood transcriptome at or after PE diagnosis

Placental single-cell signatures were evaluated at or after EOPE diagnosis using a longitudinal whole blood microarray dataset that profiled 49 controls and 13 EOPE cases, as described earlier^41^. Analysis was restricted to samples collected at 32 to 34 weeks of gestation. For each transcript cluster (i.e. gene), the effect of gestational age was removed first by subtracting the gene expression trend estimated in controls using linear mixed-effects models with non-linear terms of gestational age. These gene expression residuals were further standardized into Z-scores by subtracting the mean and dividing by the standard deviation of control samples collected ≤ 11 weeks gestation to make data among genes comparable. Cell-type signatures were calculated as the mean standardized expression of the genes in each signature in each patient sample. Discriminative performance of each single-cell signature for EOPE was evaluated within 32–34 weeks, retaining only one sample per patient. Receiver operating characteristic (ROC) curves and area under the curve (AUC) with 95% confidence intervals were calculated using the *pROC* R/Bioconductor package^137^. ROC curves were built assuming that signature scores will be higher in cases of PE than in controls.

Placental single-cell signatures were also evaluated at the time of diagnosis using another whole blood microarray dataset that included EOPE (n = 25), LOPE (n = 47), and uncomplicated pregnancies (n = 61)^42^. Controls were further stratified by gestational age at sample collection <34 weeks and ≥34 weeks to match EOPE and LOPE cases, respectively. To adjust for gestational age, probe intensities were normalized by regressing each gene on gestational age within control samples, and residuals were standardized to Z-scores by dividing by the residual standard deviation. For each sample, a signature score was computed as the mean standardized expression of the probes corresponding to the genes comprising the signature. When multiple probes mapped to the same gene, the probe with the highest coefficient of variation across samples was retained. Group comparisons of signature scores were performed using two-sample t-tests between EOPE and matched controls, and between LOPE and matched controls.

#### Evaluation of placental single-cell signatures at the protein level using maternal plasma proteomics

Proteomic validation of placental single-cell signatures was performed using a longitudinal maternal plasma dataset that measured ~7,000 proteins in 673 samples collected from 89 women with LOPE and 91 controls using an aptamer-based platform^46^. Samples were obtained at three gestational windows: T1 (15–22 weeks), T2 (22–30 weeks), and T3 (30–42 weeks). For each protein, the effect of gestational age was removed by fitting generalized additive models that included spline transformations of gestational age (basis dimension 3–6) using data from controls. The detrended data was further standardized by subtracting the mean and dividing by the standard deviation in control samples collected at T1, yielding protein-level Z-scores. Aptamers were mapped to gene symbols, and when multiple aptamers mapped to the same gene, the probe with the highest variance across samples was retained. Proteins mapping to multiple genes (e.g. protein complexes) were excluded. For each sample, a signature score was calculated as the mean standardized abundance of the proteins corresponding to genes in each signature. Differences in cell-type signature scores between cases and controls were compared using two-sample t-tests at each gestational age interval.

#### Statistical analysis of demographic data

Statistical analyses for demographic data were performed in SPSS v29.0.2 (IBM, Armonk, NY, USA). Data were compared using two-tailed Fisher’s exact tests for proportions and Mann-Whitney U-tests for non-normally distributed continuous variables.

## Extended Data

**Extended Data Fig. 1.** Quality metrics and marker gene expression in nonimmune and immune cell types. (**a**) Quality metrics for single-cell RNA-Seq data showing the number of reads per cell type, the number of features per cell type, and mitochondrial reads per cell type and (**b**) marker gene expression.

**Extended Data Fig. 2.** Marker gene expression in immune cell types.

**Extended Data Fig. 3.** Number and strength of interactions in the cell-cell communication analysis

## Supplementary Material

**Supplementary Fig. 1. Cell counts in each cell type**. Bar plots represent the numbers of each cell type with (**a**) women with PE (red bars) and controls (grey bars) and with (**b**) maternal (light blue) or fetal (dark blue) origin in the whole PE, EOPE, and LOPE.

**Supplementary Fig. 2. MA plot of all detected genes**. MA-plot showing the log2 fold changes and average expression values of differentially expressed genes (DEGs). Significant genes after p-value adjustment (FDR < 0.1) are displayed in red when they are upregulated and blue when they are down regulated in PE cases compared to controls.

**Supplementary Fig. 3. Top 25% aggregated interaction**. Bar plots represent information flow of each pathway in women with PE and controls in (**a**) whole PE, (**b**) EOPE, and (**c**) LOPE. The top 25% signaling pathways colored red are more enriched in PE, the ones colored black are equally enriched in PE and controls, and the ones colored grey are more enriched in controls.

**Supplementary Fig. 4. Placental single-cell signatures detected in the maternal circulation of normotensive pregnant women.** (**a**) Schematic overview of the integrative analysis between placental single-cell signatures and whole-blood transcriptomic profiles from maternal peripheral blood collected throughout normal pregnancy (n = 49). Line plots show the longitudinal trajectories of single-cell signatures derived from (**b**) whole PE, (**c**) EOPE, and (**d**) LOPE in the maternal circulation. Dots represent gene expression values detrended for gestational age, and colored lines indicate the average trajectories of the aggregated signatures for each cell type.

**Supplementary Fig. 5. Placental single-cell signatures detected in maternal circulation at PE diagnosis**. (**a**) Schematic overview of the integrative analysis between placental single-cell signatures and whole-blood transcriptomic profiles from maternal peripheral blood collected at the time of EOPE (n = 25) and LOPE diagnosis (n = 47). ROC curves of (**b**) whole PE, (**c**) EOPE, and (**d**) LOPE-derived signatures evaluated for their performance in differentiating EOPE and LOPE cases from matched controls. AUC values with 95% confidence intervals are reported for each signature. An AUC lower bound >0.5 indicates significant discriminatory performance.

**Supplementary Fig. 6. Correlation of DEGs between bulk and scRNA-seq analyses**. Correlation coefficient (r) and statistical significance (p) were calculated via spearman correlation analysis. Each dot represents DEGs identified in both bulk and scRNA-seq analyses.

Supplementary Table 1. Previous studies for human placental scRNA-seq studies in PE

Supplementary Table 2. Clinical characteristics of early-onset preeclampsia (EOPE) and late-onset preeclampsia (LOPE) patients and non-preeclamptic controls

Supplementary Table 3. Enrichment analysis results from GO, KEGG, and Reactome DBs

Supplementary Table 4. Placental signatures utilized for the integrative analysis

Supplementary Table 5. Placental signatures in maternal circulation during normotensive pregnancy

Supplementary Table 6. Placental signatures in maternal circulation at or after EOPE diagnosis

Supplementary Table 7. Placental signatures in maternal circulation at the time of EOPE and LOPE diagnosis

Supplementary Table 8. Protein-level abundance corresponding to cell type signatures

## Data Availability

The scRNA-seq data generated in this study have been deposited in GEO and dbGAP and are publicly available from the date of publication (dbGaP ID: phs001886.v6). Previously published sequencing data that were utilized here are available under the accession codes GSE65866, GSE65940, and GSE66273 (bulk transcriptome studies utilized in Supplementary Fig. 6). All other data needed to evaluate the conclusions in the paper are present in the paper or supplementary materials.
